# The complete mitochondrial genome of *Spondylis buprestoides* (Coleoptera: Cerambycidae)

**DOI:** 10.1080/23802359.2020.1835577

**Published:** 2020-11-20

**Authors:** Bairong Lin, Yunzhu Sun, Jiayi Ma, Rong Deng, Liangjing Sheng, Xiong Guan

**Affiliations:** a Key Laboratory of Biopesticide and Chemical Biology (Ministry of Education), College of Plant Protection, Fujian Agriculture and Forestry University, Fuzhou, PR China; b College of Forestry, Fujian Agriculture and Forestry University, Fuzhou, PR China; c Key Laboratory of Integrated Pest Management in Ecological Forests, Fujian Province University, Fujian Agriculture and Forestry University, Fuzhou, PR China

**Keywords:** Complete mitochondrial genome, *Spondylis buprestoides*, phylogenetic analysis

## Abstract

*Spondylis buprestoides* is a major boring pest of Cerambycidae, which mainly damaging conifers and also can carry pine wood nematode, *Bursaphelenchus xylophilus*. In this study, the complete mitochondrial genome of *S. buprestoides* was determined by Illumina sequencing technology. The whole mitogenome was 15,837 bp in length with 20.05% GC content, which contains 13 protein-coding genes (PCGs), 22 transfer RNA genes (tRNAs), and 2 ribosomal RNA genes (rRNAs). Phylogenetic analysis showed that *S. buprestoides* was closely related to Lepturinae. The sequence data of *S. buprestoides* could provide useful genetic information for the studies on phylogenetic and evolutionary of Cerambycidae.

Cerambycidae insects possess species richness, morphological diversity, ecological diversity, and other variable biological features, which makes the genetic evolution and classification system of this group attract extensive attention (Zhang et al. 2018; Wang et al. 2019). *Spondylis buprestoides* is a major boring pest of Cerambycidae, which mainly damaging conifers such as *Pinus massoniana* Lamb., *P. tabulaeformis* Carr., *P. armandi* Franch., etc., and also can carry pine wood nematode, *Bursaphelenchus xylophilus* (Zhou et al. 2012). However, there are only few reports about the genetic evolution analysis of *S. buprestoides*. In this study, we reported the complete mitochondrial genome of *S. buprestoides* based on Illumina sequencing data and investigated the phylogenetic relationship by maximum-likelihood tree inference method.

The samples of *S. buprestoides* were collected from Minhou (119° 22′ 53′′E, 25° 51′ 17′′N), Fujian Province, China, and the voucher specimens were deposited in the Fujian Agriculture and Forestry University (TN-202006). The total DNA extraction from *S. buprestoides* by TruSeq DNA Sample Preparation kit (Vanzyme, Nanjing, China). And the purification DNA was used to construct library with 300 bp randomly interrupted fragment. The constructed library was quantified by Qubit (Thermo Fisher Scientific Inc., Waltham, MA), and then was sequenced through the Illumina Hiseq2500 platform. A total of 58,949,412 clean reads were obtained after filtering by Fastp software (Chen et al. 2018). The clean reads were assembled by MtioZ and metaSPAdes software (Nurk et al. 2017). And the assembly sequence was annotated by the MITOS web server (Matthias et al. 2013) with *Aristobia reticulator* (GenBank accession no. MK423971) as the reference. The complete mitochondrial genome of *S. buprestoides* (GenBank accession no. MT826860) forms a circular structure covering 15,837 bp in length, with 13 protein-coding genes (PCGs), 22 transfer RNA genes (tRNAs), and 2 ribosomal RNA genes (rRNAs). The A + T content in the mtDNA of *S. buprestoides* was 79.95%, within the range from 67.1% to 80.8% of beetle mtDNA (Kim et al. 2009), and the A + T content of PCGs, tRNAs, and rRNAs was 77.59%, 79.33%, and 82.86%, respectively. The mitochondrial composition of *S. buprestoides* was identical to the case of other Cerambycidae.

With the aim to further reveal the phylogenetic position of *S. buprestoides*, the phylogenetic relationships were constructed of *S. buprestoides* based on the other 23 different species of Cerambycidae, which multiple aligned by MUSCLE version 3.8 (Edgar 2004), and then the Maximum-Likelihood tree was inferred by IQ-TREE version 1.6.11 (Nguyen et al. 2015) with the parameter of -bb 1000. Phylogenetic analysis results supported that *S. buprestoides* was in close association with *Anastrangalia sequensi* (Lepturinae) (Figure 1). In summary, the complete mitochondrial genome of *S. buprestoides* will provide useful genetic information for increasing the richness of the Cerambycidae, as well as assisting in phylogenetic and evolutionary studies of Cerambycidae.

**Figure 1.**
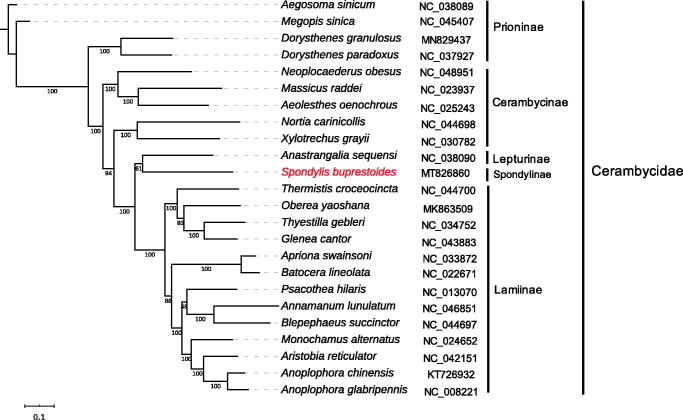
Phylogenetic relationships of Cerambycidae by maximum-likelihood tree inference method. The data contains 24 different species of Cerambycidae. Bootstrap support values are labeled near the branch.

## Data Availability

The data supporting the findings of this study are openly available in GenBank of NCBI at https://www.ncbi.nlm.nih.gov. The complete mitochondrial genome of *Spondylis buprestoides* is available in the NCBI with the accession number MT826860, and the Illumina sequencing data are available in the NCBI database via the BioProject number PRJNA662866.

## References

[CIT0001] Chen SF, Zhou YQ, Chen Y, Gu J. 2018. fastp: an ultra-fast all-in-one FASTQ preprocessor. Bioinformatics. 34(17):i884–i890. 30423086 10.1093/bioinformatics/bty560 PMC6129281

[CIT0002] Edgar RC. 2004. MUSCLE: multiple sequence alignment with high accuracy and high throughput. Nuclc Acids Res. 32(5):1792–1797. 10.1093/nar/gkh340 PMC390337 15034147

[CIT0003] Kim KG, Hong YM, Kim MJ, Im H, Kim MI, Bae CH, Seo SJ, Lee SH, Kim I. 2009. Complete mitochondrial genome sequence of the yellow-spotted long-horned beetle *Psacothea hilaris* (Coleoptera: Cerambycidae) and phylogenetic analysis among coleopteran insects. Mol Cells. 27(4):429–441. 19390824 10.1007/s10059-009-0064-5

[CIT0004] Matthias B, Alexander D, Frank J, Fabian E, Catherine F, Guido F, Joern P, Martin M, Peter FS. 2013. MITOS: improved de novo metazoan mitochondrial genome annotation. Mol Phylogenet Evol. 69(2):313–319. 22982435 10.1016/j.ympev.2012.08.023

[CIT0005] Nguyen LT, Schmidt H, von Haeseler A, Minh B. 2015. IQ-TREE: a fast and effective stochastic algorithm for estimating maximum-likelihood phylogenies. Mol Biol Evol. 32(1):268–274. 25371430 10.1093/molbev/msu300 PMC4271533

[CIT0006] Nurk S, Meleshko D, Korobeynikov A, Pevzner PA. 2017. metaSPAdes: a new versatile metagenomic assembler. Genome Res. 27(5):824–834. 28298430 10.1101/gr.213959.116 PMC5411777

[CIT0007] Wang J, Dai XY, Xu XD, Zhang ZY, Yu DN, Storey KB, Zhang JY. 2019. The complete mitochondrial genomes of five longicorn beetles (Coleoptera: Cerambycidae) and phylogenetic relationships within Cerambycidae. PeerJ. 7(2):e7633. 31534857 10.7717/peerj.7633 PMC6732212

[CIT0008] Zhang SQ, Che LH, Li Y, Liang D, Pang H, Slipinski A, Zhang P. 2018. Evolutionary history of Coleoptera revealed by extensive sampling of genes and species. Nat Commun. 9(1):205. 29335414 10.1038/s41467-017-02644-4 PMC5768713

[CIT0009] Zhou SY, Chen HH, Xiang NY. 2012. Spatial pattern and time series dynamics of *Spondylis buprestoides* adults. Plant Dis Pests. 3(2):38–41.

